# Determination of the sensitivity and specificity of bovine tuberculosis screening tests in dairy herds in Thailand using a Bayesian approach

**DOI:** 10.1186/s12917-019-1905-x

**Published:** 2019-05-16

**Authors:** Tawatchai Singhla, Sukolrat Boonyayatra, Songkhla Chulakasian, Mintra Lukkana, Julio Alvarez, Srinand Sreevatsan, Scott J. Wells

**Affiliations:** 1 Department of Food Animal Clinic and Excellent Center of Veterinary Public Health, Faculty of Veterinary Medicine, Chiang Mai University, Chiang Mai, Thailand; 2 National Bureau of Agricultural Commodity and Food Standards, Bangkok, Thailand; 3 Department of Veterinary Population Medicine, College of Veterinary Medicine, University of Minnesota, St. Paul, MN USA; 4 Center for Animal Health and Food Safety, University of Minnesota, St. Paul, MN USA

**Keywords:** *Mycobacterium bovis*, Test performance, Single intradermal tuberculin test, Interferon gamma assay, ELISA test

## Abstract

**Background:**

The objective of this study was to determine the sensitivity (Se) and specificity (Sp) of bovine tuberculosis (bTB) screening tests including a single intradermal tuberculin (SIT) test, interferon gamma (IFN-γ) assay, and a commercial ELISA test (*M. bovis* Ab) in dairy cattle, under field conditions, using a Bayesian approach.

**Results:**

The study population consisted of 128 dairy cows from 25 bTB-infected herds in Chiang Mai and Chiang Rai provinces, Thailand. A single-population Bayesian model was implemented assuming conditional dependence between the SIT test and IFN-γ assays. The 95% posterior probability interval (PPI) of the SIT test (severe interpretation) Se ranged from 75.3 to 95.2% (median = 87.6%), while the Sp was slightly lower (median = 83.6%, PPI = 74.2–92.8%). The IFN-γ assay Se was moderate and the 95% PPI ranged from 38.6 to 74.4% (median = 55.7%) with higher Sp (median = 93.5.4%, PPI = 87.0–98.1%). The *M. bovis* Ab ELISA Se was low, with 95% PPI ranging between 30.0 and 71.2% (median = 47.4%); however, the Sp was high (median = 90.9%, PPI = 84.5–95.5%).

**Conclusion:**

The SIT test sensitivity was similar to that demonstrated in other regions and can, therefore, be used effectively as part of control programs in this area. The IFN-γ and *M. bovis* Ab ELISA assays can be applied as supplementary techniques. The test performance of these tests when used as single tests without confirmation, however, are expected to continue to challenge disease eradication efforts.

**Electronic supplementary material:**

The online version of this article (10.1186/s12917-019-1905-x) contains supplementary material, which is available to authorized users.

## Background

Bovine tuberculosis (bTB) is a chronic endemic disease of cattle and other ruminants. The disease is primarily caused by *Mycobacterium bovis*, which affects animal health and can spill over to humans as a zoonotic disease [1]. Test-and-cull strategies have been applied in cattle globally; however, the disease is still prevalent in many countries. The success of bTB eradication and control programs is based on early detection and removal of reactors from a herd. Therefore, screening-test accuracy is critical to eradication programs. However, the lack of a reliable gold standard to define positive and negative individuals is a problem in determining the accuracy of any screening test. In other countries with abattoir surveillance, the gold standard for diagnosis of bovine tuberculosis is based on bacterial culture or PCR but it is not feasible in countries without abattoir surveillance to identify lesions.

The single intradermal tuberculin (SIT) test, based on the detection of the cell-mediated immune response (CMI), is used for bTB diagnosis worldwide. The SIT test is performed by inoculating bovine purified protein derivative (PPD) into the skin of the neck or caudal fold of the animal. Its interpretation is based on measuring the difference in skin thickness before and after inoculation [2]. The interferon-gamma detection test (IFN-γ assay) is a blood-based assay that also detects the CMI in blood samples stimulated with specific antigens [3]. This method is widely used in many countries; however, the practical use of this test is limited by the need for processing blood samples within 24 h of collection. As a result, the IFN-γ assay cannot be applied at a large scale or in herds located in remote areas where farms are situated far from specialized laboratories [4, 5]. Serological tests based on antibody detection are another option for bTB screening and can identify *M. bovis*-infected cattle missed by current bTB screening techniques. One commercially available enzyme-linked immunosorbent assay (ELISA) that detects antibodies against *M. bovis* antigens MPB83 and MPB70 (*M. bovis* Ab ELISA) in naturally infected cattle has been used and its performance determined [6]. However, the sensitivity and specificity of these screening tests appear to vary [7–11].

Latent class analysis is being increasingly applied for the estimation of screening test performance in many diseases and species when a reference test (a gold standard) is scarce [12, 13]. Bayesian latent class analysis has been used to evaluate the accuracy of two or more bTB screening tests in cattle without the use of a reference test when the true disease status is unknown [7, 14]. Nevertheless, to the authors’ knowledge, the evaluation of bTB screening-test performance using latent class models has never been performed in Southeast Asia.

The objective of this study was to evaluate the Se and the Sp of currently available official bTB screening tests in Thailand (SIT test and IFN-γ assay) and the ancillary test (*M. bovis* Ab ELISA) in dairy cows under field conditions using a Bayesian approach.

## Methods

### Study and sampling design

This study population was dairy cattle from 25 herds with previous SIT-positive cattle in Chiang Mai (16 farms) and Chiang Rai (9 farms) provinces. These herds were previously considered as bTB infected herds based on the presenting of at least one SIT-positive animal in the farms during 2011 to 2015. In these herds, the median number of cows in each dairy herd was 53 (interquartile range = 33–67). From each of these 25 herds, approximately 5–6 animals were selected as the sample population from which the 3 bTB diagnostic tests were performed. SIT-positive animals as regards to the bTB annual testing performed in 2015 were primarily selected to be included in the study. Other SIT-negative animals within each farms were randomly selected to make the sample of 5–6 animals per herd.

### SIT test

All adult dairy cattle (> 1 year old) in each herd were tested using the caudal fold SIT test by one of the authors or Thai Department of Livestock and Development (DLD) staffs using bovine PPD (Bovituber® PPD, Synbiotics, Lyon, France) between March and May 2015. The dairy cows were intradermally injected with 0.1 mL of bovine PPD (2000 IU) on the right side of the caudal fold of the tail. The skin thickness of the inoculation site was measured using calipers before injection. Test results were determined by the same researcher at 72 h post-injection by measuring the increase in skinfold thickness. Interpretations of the test results were made according to the Thai agricultural standard for screening tests for bovine tuberculosis [2]. The results were defined as: positive when the increase of the skinfold thickness at the inoculation site was ≥5 mm and/or signs of swelling, edema, exudation, necrosis and/or inflammation were observed; inconclusive when the increase of the skinfold thickness was between 2 and 5 mm and clinical signs at the inoculation site were not observed; and negative when the skinfold thickness increased < 2 mm and clinical lesions at the injection site were not observed. Depending on the interpretation used, inconclusive animals were considered as positive (severe interpretation) or negative (standard interpretation) for data analysis.

### Interferon gamma assay

At 72 h post-bovine PPD injection, heparinized blood samples were collected from all SIT-tested dairy cows and transported to the laboratory of the Northern Veterinary Research and Development Center, Upper zone, Lampang, Thailand, where they were routinely processed within 6 h [4, 5]. Stimulation of whole-blood samples was performed as described elsewhere [8]. Briefly, whole-blood samples were separated into three parts and each was incubated with bovine PPD, avian PPD, and phosphate buffered saline (PBS). After 16–24 h of incubation, plasma supernatants were harvested and IFN-γ quantified using a commercially available sandwich ELISA (Thermo Fisher Scientific, Waltham, MA, USA). Optical density (OD) was measured on each sample stimulated with bovine PPD, avian PPD, and PBS. As recommended by the manufacturer, a sample was considered as a positive sample when both 1) the difference between the mean OD of the sample stimulated with bovine PPD and with PBS alone, and 2) the difference between the mean bovine PPD and avian PPD-stimulated sample ODs were greater than 0.1 [8].

### Antibody detection test (*M. bovis* ab ELISA)

At the time of bovine PPD inoculation, serum samples from all dairy cows were collected and tested using a commercial ELISA kit (IDEXX *M. bovis* Ab test, IDEXX Laboratories Inc., Westbrook, ME) in accordance with the manufacturer’s instructions. Results are presented as sample-to-positive ratios (S/P) using corrected ODs. Samples with S/P ratios ≥0.30 were considered positive [6].

### Sensitivity (Se) and specificity (Sp) estimation

A Fleiss kappa analysis was conducted to assess the agreement between the three screening tests results. This method was applied for analysis of agreement between more than two raters [15]. Analysis results were categorized into six categories based on kappa values (< 0–1): poor, slight, fair, moderate, substantial, and almost perfect agreement [16].

A latent class analysis was performed using a Bayesian model to estimate Se and Sp of the SIT test, the IFN-γ assay, and the *M. bovis* Ab ELISA. As the SIT test and the IFN-γ assay share similar principles for detection of the cellular immune response, their results were considered to be conditionally dependent on each other [17]. In contrast, the *M. bovis* Ab ELISA is based on detection of the humoral immune response and therefore test results were assumed to be conditionally independent of both SIT and IFN-γ assays. Thus, a Bayesian model allowing conditional covariance between the SIT and IFN-γ assays given infection status, while assuming both SIT and IFN-γ assays conditionally independent of the *M. bovis* Ab ELISA, was also specified. The samples were randomly selected from the SIT-positive herds which located in the same area. Therefore, it could be assumed that the sample were from the same population as suggested in a study in Spain [7]. Thus, a Bayesian model for two conditionally dependent tests and one conditionally independent test was implemented in a single population in the order to evaluate Se and Sp of each test.

The Bayesian version of the latent class model assumed that for the k populations, the counts (Y_*k*_) of the different combinations of test results, e.g. +/+/+, +/+/−, etc. for three tests follow a multinomial distribution: Y_*k*_ | P_*qrsk*_ ~ multinomial (n_*k*_, {P_*qrsk*_}), where *qrs* was the multinomial cell probability for the three-test outcome combination, and P_*qrsk*_ was a vector of probabilities of observing the individual combinations of test results. A complete model and the R codes are provided in the Additional file 1. Prior information on the test performance and prevalence of the disease was introduced in the analysis using probability distributions (prior distributions). Prior Se and Sp estimates of the Sp of the three tests were modeled as beta distributions based on information obtained from previous studies [3, 7, 9–11, 14, 18–20]. Published study means of the central values were selected as the most likely value, while a 95% lower limit for the prior distributions was set using the lowest modal value to accommodate the expected large variability in test performance. Prior bTB prevalence rates were selected based on a report from the DLD and expert opinion from the official veterinary services managing the bTB eradication programs in Chiang Mai, Chiang Rai, and Phayao provinces [21]. The prior values used for analysis (prevalence, sensitivity, specificity) are listed in Table 1. All analyses were implemented in JAGS 3. 4. 0 via the rjags and R2jags packages from R 3.2.2 software [22–24]. Posterior distributions were computed after 100,000 iterations of the models with the first 10,000 discarded as the burn-in phase.

**Table 1:** Prior mode and 95% confidence interval (CI) estimates for each screening test

Parameter	Test	Mode	95% CI	References
Prevalence		10	< 20.0	[21]
Sensitivity	SIT (standard)^a^	71	> 53.2	[9, 20]
	SIT (severe)^b^	81	> 63.2	[9, 11, 27]
	IFN-γ ^c^	81.9	> 64.1	[3, 7, 14, 18]
	ELISA^d^	60	> 34.4	[6, 18]
Specificity	SIT (standard)^a^	98.6	> 89.2	[9, 20]
	SIT (severe)^b^	95.6	> 89.2	[9, 11, 27]
	IFN-γ ^c^	87.9	> 70.0	[3, 7, 14, 18]
	ELISA^e^	86.5	> 75.0	[6, 18]

^a^ single intradermal tuberculin test using standard interpretation

^b^ single intradermal tuberculin test using severe interpretation

^c^ interferon gamma assay

^d^ a commercial antibody detection test (*M. bovis* Ab ELISA)

Convergence of the model was checked by visual inspection of the Gelman-Rubin diagnostic plots using three sample chains with different initial values as demonstrated in Additional file 2 [25]. A sensitivity analysis of the model was performed to assess the influence of the prior information and the assumption of conditional dependence between the SIT test and the IFN-γ assay on the posterior estimates [12, 13]. These analyses were performed by replacing each prior by a non-informative uniform 0–1 distribution and comparing the DIC between the models with and without the covariance term [13].

## Results

### Results from screening tests

Twenty-four and 54 of the 128 dairy cows (18.75 and 42.19%) were positive based on the SIT test using the standard and the severe interpretation, respectively. Herd prevalence of bTB based on the standard and the severe interpretation of the SIT test among sampled cows in these herds was 36% (9/25) and 60% (15/25), respectively. Compared to the SIT test, fewer positive results were detected among dairy cows using the IFN-γ assay (22/128 = 17.19%) and the *M. bovis* Ab ELISA (21/128 = 16.40%) (Table 2). The agreement between the SIT test using standard interpretation and the IFN-γ assay was fair (kappa = 0.21). The agreement between the SIT test using standard interpretation and the *M. bovis* Ab ELISA was slight (0.11). Similarly, the agreement between the IFN-γ assay and the *M. bovis* Ab ELISA was also slight (0.13). The agreement between all three tests, SIT test using standard interpretation, IFN-γ assay, and *M. bovis* Ab ELISA, was slight (kappa = 0.15), whereas the agreement between the SIT test using severe interpretation, the IFN-γ assay, and the *M. bovis* Ab ELISA was fair (kappa = 0.24).

**Table 2:** Screening test results in dairy cows

SIT^a^	IFN-γ ^b^	ELISA^c^	SIT (standard)^d^	SIT (severe)^e^
+	+	+	0	5
+	+	–	8	15
+	–	+	6	11
+	–	–	10	23
–	+	+	6	1
–	+	–	8	1
–	–	+	9	4
–	–	–	81	68
Total			128	128

^a^ single intradermal tuberculin test using standard interpretation

^b^ interferon gamma assay

^c^ a commercial antibody detection test (*M. bovis* Ab ELISA)

^d^ single intradermal tuberculin test using standard interpretation

^e^ single intradermal tuberculin test using severe interpretation

### Bayesian models

SIT-test Se estimates were 62.4 and 87.6%, when standard and severe interpretations were applied, respectively. The SIT Se using standard interpretation was lower than the prior estimate, while the SIT Se using severe interpretation was higher. However, the probability intervals of these Se estimates did not overlap, when comparing prior to posterior estimates. The SIT-test Sp estimates were lower than the prior estimates regardless of standard (90.6%) and severe interpretations (83.6%). Posterior estimates of the SIT test Se and Sp are shown in Tables 3 and 4.

**Table 3:** Bayesian estimates of sensitivity and specificity for each test, and disease prevalence (%)

Parameter	Test	Median	95% PPI^a^
Sensitivity	SIT (standard)^b^	62.4	43.2–80.2
	IFN-γ ^c^	60.1	39.8–81.3
	ELISA^d^	52.5	30.8–77.7
Specificity	SIT (standard)^b^	90.6	82.9–97.6
	IFN-γ ^c^	89.0	81.6–95.4
	ELISA^d^	88.3	81.8–93.3
Disease prevalence		14.1	6.7–23.4
Covariance of infected cattle		−9.9	−22.3-2.9
Covariance of uninfected cattle		2.6	−0.3-6.7

^a^ 95% PPI = 95% posterior probability interval

^b^ single intradermal tuberculin test using standard interpretation

^c^ interferon gamma assay

^d^ a commercial antibody detection test (*M. bovis* Ab ELISA)

**Table 4:** Bayesian estimates of sensitivity and specificity for each test, and disease prevalence (%)

Parameter	Test	Median	95% PPI^a^
Sensitivity	SIT (severe)^b^	87.6	75.3–95.2
	IFN-γ ^c^	55.7	38.6–74.4
	ELISA^d^	47.4	30.0–71.2
Specificity	SIT (severe)^b^	83.6	74.2–92.8
	IFN-γ ^c^	93.5	87.0–98.1
	ELISA^d^	90.9	84.5–95.5
Disease prevalence		22.2	11.9–33.6
Covariance of infected cattle		−1.2	−7.3-6.0
Covariance of uninfected cattle		3.4	0.0–8.0

^a^ 95% PPI = 95% posterior probability interval

^b^ single intradermal tuberculin test using severe interpretation

^c^ interferon gamma assay

^d^ a commercial antibody detection test (*M. bovis* Ab ELISA)

Se estimates for IFN-γ and *M. bovis* Ab ELISA were lower than the prior values. Posterior estimates of Sp for both techniques were higher than the prior estimates (Tables 3 and 4). The IFN-γ assay outperformed the *M. bovis* Ab ELISA in terms of Se, although probability intervals for the posterior estimates largely overlapped. A very similar (and high) specificity was found for both tests.

Posterior prevalence estimates in dairy cattle were higher than the prior estimates and varied depending on the interpretation criteria used, with median values ranging from 14.1% (standard interpretation) to 22.2% (severe interpretation) (Tables 3 and 4).

The conditional covariance between the SIT test and the IFN-γ assay was low in both infected and uninfected cattle. Probability intervals of the conditional covariance included 0 regardless of the interpretation criteria for the SIT test. The conditional independent model, which did not include a covariance term between the SIT test and IFN-γ assay, had a higher DIC value than the conditional dependent model (66.2 versus 46.7, respectively). Therefore, the conditional dependent model was preferred as the final model.

There was no appreciable effect on sensitivity analyses (change > 25% of median value) in the posterior estimates of the *M. bovis* Ab ELISA Se, and the Sp of all three screening tests when non-informative distributions were used as priors for any parameter. For instance, the posterior estimate of the *M. bovis* Ab ELISA Se changed by only 2.7% (from 47.4 to 48.7%) when non-informative distribution was used. This finding was interpreted as evidence of model robustness. In contrast, a larger change in the posterior estimates for the SIT-test using standard interpretation (from 62.4 to 31.2%) and the IFN-γ assay Se (from 60.1 to 27.4%) was observed. Similarly, the prevalence estimate in the dairy cattle population also increased to 20.5% [95% posterior probability interval (PPI) = 7.2–33.2%] when a non-informative prior was used, thus, suggesting a stronger effect for these parameter priors in the model.

## Discussion

This study assessed the performance of bTB screening tests routinely used in eradication programs (SIT test and IFN-γ assay) and a potential supplementary test (*M. bovis* Ab ELISA) under field conditions in Thailand using a Bayesian approach. A one-population model was chosen for the analysis because the screening tests were performed in infected dairy herds located in the same region and followed similar management practices. Therefore, considering all dairy cattle as a single population was reasonable, as assumed in previous studies [7, 14].

The fair agreement between the three tests using Fleiss’ kappa was similar to the agreements between two tests using Cohen’s kappa analysis. The lack of correlation between the test outcomes suggests that their application as parallel tests would help to increase the performance of the screening strategy in current bTB eradication programs [8].

The median SIT-test Se using a standard interpretation in our study was similar to that reported in a study in Australia in 1991 (63.2%); this country reported low bTB prevalence at the time and was recognized as free from bTB in 1997 [20]. The estimated SIT Se using severe interpretation was similar to results from a US study showing a SIT-test Se range of 84.9–93.02% [11]. Several studies have reported that both the size of the skin-test response and the pathological lesions are positively associated with the infection stage [8, 26]. In Thailand, limited information is currently available to estimate the prevalence of bTB in dairy cattle. One government report [21] reported the prevalence of bTB among dairy cattle in northern Thailand to be 0.30 and 4.38% at the animal-level and the herd-level, respectively, based on SIT testing. In our study, our posterior estimate of true prevalence of bTB was 14–22%, depending on SIT test interpretation method, higher than previously reported, though was from a biased sample of cattle from test-positive herds.

In Thailand, the SIT test is performed annually together with culling of reactors in all infected herds. Thus, infected animals with advanced infections are quite rare, which could reduce the SIT-test Se using the standard interpretation (inconclusive results defined as negative). The SIT-test Sp in the current study was similar to those reported in previous studies in low prevalence areas, which ranged from 83.6 to 90.6% [10, 20] though lower than Sp reported from a meta-analysis of US studies [9]. In Thailand, confirmatory testing of SIT-positive cattle or surveillance at abattoirs is not performed.

Estimates for the Se of the IFN-γ assay were also lower than reported in previous studies [5, 20]. It has been suggested that the IFN-γ assay should be performed between 7 and 33 days after the SIT test to maximize the effect [5]. However, in the current study, we collected whole-blood samples for the IFN-γ assay only three days after performing the SIT test due to the limitations of time and labor. This early blood collection might impair the performance of the IFN-γ in this study. However, Whipple et al. (2001) reported that the SIT test boosted the IFN-γ responses three days after tuberculin injection, and the USA Department of Agriculture recommends applying the test from 3 to 30 days after the SIT test [27].

Our estimates for the IFN-γ assay Sp were high, which is in agreement with previous studies [3]. However, a study on the performance of the IFN-γ assay and the SIT test under field conditions in France reported a more limited IFN-γ assay Sp estimate of 62.3% [28].

Our posterior estimates of the *M. bovis* Ab ELISA Se were lower than previous reports in Spain and the USA [6, 29]. In the US study, the *M. bovis* Ab ELISA was applied to test *M. bovis* challenged calves, and the test Se was 63.0%. However, the test Se decreased to 46.0% when applied to cattle without pathogenic lesions [6]. A study in Spain suggested that *M. bovis* Ab ELISA Se could be maximized up to 70.4% when the test was applied to the infected herd 15 days after the SIT test by taking advantage of anamnestic effect [29]. Moreover, the Se of the test could be as low as 23.9% when the test was performed in naturally infected herds without previous SIT test boosting [29]. In the current study, most blood samples were collected without a previous SIT test boost. However, posterior estimates of the *M. bovis* Ab ELISA Sp were high, in agreement with previous studies [6, 29].

Overall, based on study estimates of the performance of the bTB diagnostic assays (SIT test, IFN-γ assay, and ELISA), we can make several general conclusions. First, since the prevalence of bTB in this region appears higher than that in the US, Western Europe, and Australia (where most published reports of bTB test performance have been generated), we would expect higher positive predictive values and lower negative predictive values with the same tests, given the same test performance. However, this study reports a lower test sensitivity for the SIT test (using standard interpretation) than that reported from most previous studies. In order to increase test sensitivity, the severe interpretation could be used, though this would reduce test specificity (and positive predictive value) to a level that may be less conducive by itself for test and removal programs. Use of the standard interpretation of the SIT test instead would increase test specificity, but at the costs of reduced test sensitivity. Surveillance system sensitivity could be increased through use of tests in combination (at increased costs), or through incorporation of abattoir surveillance (with confirmatory testing, also at higher costs). Evaluation of the cost-effectiveness of alternative surveillance system strategies is a next step, and certainly warranted by study findings.

## Conclusion

This study provides estimates of the sensitivity and specificity of currently available tests for bTB screening in Thailand (SIT test and IFN-γ assay) and an ancillary test (*M. bovis* Ab ELISA) in dairy cows, under field conditions, using a Bayesian approach. This information is critical to effective bTB control and eradication programs in Thailand and across Southeast Asia. However, low number of positive results limits the test performance estimation. Therefore, a future study should be performed in larger dairy cattle population or areas.

## Additional files

Additional file 1: Two conditionally dependent tests and one conditionally independent test, one population model. (DOCX 13 kb)

Additional file 2: Checking of convergence of two conditionally dependent and one conditionally dependent with one population model using Gelman-Rubin diagnostic plots (DOCX 926 kb)

## Abbreviations

bTB: bovine tuberculosis
CMI: cellular mediated immune response
DLD: Thai Department of Livestock and Development
ELISA: enzyme-linked immunosorbent assay
G-IFN: gamma interferon assay
*M. bovis* Ab ELISA: a commercial antibody detection test
OD: optical density
PBS: phosphate buffered saline
PPD: purified protein derivative
Se: sensitivity
SIT: single intradermal tuberculin test
Sp: specificity
TMB: tetra-methyl-benzidine

## References

[1] Ayele WY, Neill SD, Zinsstag J, Weiss MG, Pavlik I (2004). Bovine tuberculosis: an old disease but a new threat to Africa. Int J Tuberc Lung Dis.

[2] Diagnostic test of bovine tuberculosis. Bangkok: the Royal Gazette. In: Ministry of Agricultural and Cooperatives; 2004.

[3] Antognoli MC, Remmenga MD, Bengtson SD, Clark HJ, Orloski KA, Gustafson LL (2011). Analysis of the diagnostic accuracy of the gamma interferon assay for detection of bovine tuberculosis in US herds. Prev Vet Med..

[4] Terrestrial Manual OIE. Chapter 2.4.7: Bovine. tuberculosis. 2009; http://www.oie.int/fileadmin/Home/eng/Health_standards/tahm/3.04.06_BOVINE_TB.pdf. Accessed 11 Aug 2016.

[5] Ryan TJ, Buddle BM, De Lisle GW (2000). An evaluation of the gamma interferon test for detecting bovine tuberculosis in cattle 8 to 28 days after tuberculin skin testing. Res Vet Sci.

[6] Waters WR, Buddle BM, Vordermeier HM, Gormley E, Palmer MV, Thacker TC (2011). Development and evaluation of an enzyme-linked immunosorbent assay for use in the detection of bovine tuberculosis in cattle. Clin Vaccine Immunol.

[7] Alvarez J, Perez A, Bezos J, Marqués S, Grau A, Saez JL (2012). Evaluation of the sensitivity and specificity of bovine tuberculosis diagnostic tests in naturally infected cattle herds using a Bayesian approach. Vet Microbiol.

[8] de la Rua-Domenech R, Goodchild AT, Vordermeier HM, Hewinson RG, Christiansen KH, Clifton-Hadley RS (2006). Ante mortem diagnosis of tuberculosis in cattle: a review of the tuberculin tests, γ-interferon assay and other ancillary diagnostic techniques. Res Vet Sci.

[9] Farnham MW, Norby B, Goldsmith TJ, Wells SJ (2012). Meta-analysis of field studies on bovine tuberculosis skin tests in United States cattle herds. Prev Vet Med..

[10] Norby B. Performance of bovine tuberculin skin tests and factors affecting the proportion of false positive skin test results in Michigan. Ph.D. Thesis. Michigan State University: East Lansing MI;2003.

[11] Norby B, Bartlett PC, Fitzgerald SD, Granger LM, Bruning-Fann CS, Whipple DL (2004). The sensitivity of gross necropsy, caudal fold and comparative cervical tests for the diagnosis of bovine tuberculosis. J Vet Diagn Investig.

[12] Rahman AK, Saegerman C, Berkvens D, Fretin D, Gani MO, Ershaduzzaman M (2013). Bayesian estimation of true prevalence, sensitivity and specificity of indirect ELISA, rose bengal test and slow agglutination test for the diagnosis of brucellosis in sheep and goats in Bangladesh. Prev Vet Med.

[13] Branscum AJ, Gardner IA, Johnson WO (2005). Estimation of diagnostic-test sensitivity and specificity through Bayesian modeling. Prev Vet Med..

[14] Clegg TA, Duignan A, Whelan C, Gormley E, Good M, Clarke J (2011). Using latent class analysis to estimate the test characteristics of the g-interferon test, the single intradermal comparative tuberculin test and a multiplex immunoassay under Irish conditions. Vet Microbiol.

[15] Fleiss JL (1971). Measuring nominal scale agreement among many raters. Psychol Bull.

[16] Landis JR, Koch GG (1977). The measurement of observer agreement for categorical data. Biometrics.

[17] Gardner IA, Stryhn H, Lind P, Collins MT (2000). Conditional dependence between tests affects the diagnosis and surveillance of animal diseases. Prev Vet Med..

[18] EFSAScientific opinion on the use of a gamma interferon test for the diagnosis of bovine tuberculosis. EFSA J. 2012;10:2975–3038.

[19] Bezos J, Casal C, Romero B, Schroeder B, Hardegger R, Raeber AJ (2014). Current *ante-mortem* techniques for diagnosis of bovine tuberculosis. Res Vet Sci.

[20] Wood PR, Corner LA, Rothel JS, Baldock C, Jones SL, Cousins DB (1991). Field comparison of the interferon-gamma assay and intradermal tuberculin test for the diagnosis of bovine tuberculosis. Aust Vet J.

[21] Nuamjit M, Rodtian P (2012). Prevalence of bovine tuberculosis in upper northern Thailand from December 2010 to may 2011. Northern Animal Health News.

[22] Plummer M, Stukalov A, Denwood M. Rjags: Bayesian Graphical Models using MCMC. 2016. https://cran.r-project.org/web/packages/rjags/rjags.pdf. Accessed 4 Jul 2018.

[23] Su YS, Yajima M. R2jags: Using R to Run 'JAGS'. 2015. https://cran.r-project.org/web/packages/R2jags/R2jags.pdf. Accessed 4 Jul 2018.

[24] R Core Team. R: a language and environment for statistical computing. In: R Foundation for statistical computing, Vienna, Austria. 2015. http://www.R-project.org. Accessed 4 Jul 2018.

[25] Gelman A, Rubin DB (1992). Inference from iterative simulation using multiple sequences. Stat Sci.

[26] Clifton-Hadley RS, Goodchild AV. The fall and rise of bovine tuberculosis in Great Britain. In: Thoen, C.O., Steele, J.H., Gilsdorf, M.F. (Eds.), *Mycobacterium bovis* infection in animals and humans. New York: Blackwell; 2005.

[27] Whipple DL, Palmer MV, Slaughter RE, Jones SL (2001). Comparison of purified protein derivatives and effect of skin testing on results of a commercial gamma-interferon assay for diagnosis of tuberculosis in cattle. J Vet Diagn Investig.

[28] Praud A, Boschiroli ML, Meyer L, Garin-Bastuji B, Dufour B (2015). Assessment of the sensitivity of the gamma-interferon test and the single intradermal comparative cervical test for the diagnosis of bovine tuberculosis under field conditions. Epidemiol Infect.

[29] Casal C, Díez-Guerrier A, Álvarez J, Rodriguez-Campos S, Mateos A, Linscott R (2014). Strategic use of serology for the diagnosis of bovine tuberculosis after intradermal skin testing. Vet Microbiol.

