# Supplementary material for: Determination of the sensitivity and specificity of bovine tuberculosis screening tests in dairy herds in Thailand using a Bayesian approach
Source: BMC Vet Res. 2019 May 16;15:149. doi: 10.1186/s12917-019-1905-x (PMC6524330; doi:10.1186/s12917-019-1905-x)
Supplement: Supplementary file 1 — Two conditionally dependent tests and one conditionally independent test, one population model. (DOCX 13 kb) [file 12917_2019_1905_MOESM1_ESM.docx]

**Supplementary 1:** Two conditionally dependent tests and one conditionally independent test, one population model.

library(rjags)

library(R2jags)

#Data

data<-list(n=128,y=c(0,6,6,9,8,8,10,81))

model<-function()

{

#data

y[1:8]~dmulti(p[1:8],n)

p[1:8]<-c(p111,p011,p101,p001,p110,p010,p100,p000)

p111<-pi*Se3*Se11+(1-pi)*(1-Sp3)*Sp11

p101<-pi*Se3*Se12+(1-pi)*(1-Sp3)*Sp12

p011<-pi*Se3*Se21+(1-pi)*(1-Sp3)*Sp21

p001<-pi*Se3*Se22+(1-pi)*(1-Sp3)*Sp22

p110<-pi*(1-Se3)*Se11+(1-pi)*Sp3*Sp11

p100<-pi*(1-Se3)*Se12+(1-pi)*Sp3*Sp12

p010<-pi*(1-Se3)*Se21+(1-pi)*Sp3*Sp21

p000<-pi*(1-Se3)*Se22+(1-pi)*Sp3*Sp22

pi ~ dbeta(5.619,42.571)

Se1~dbeta(16.693,7.409)

Sp1~dbeta(33.342,1.459)

Se2~dbeta(13.741,4.790)

Sp2~dbeta(17.902,3.327)

Se3~dbeta(6.685,4.790)

Sp3~dbeta(36.199,6.493)

Se11<-CovD_pos+(Se1*Se2)

Se21<-Se2-Se11

Se12<-Se1-Se11

Se22<-1-(Se11+Se12+Se21)

Sp11<-1-(Sp12+Sp21+Sp22)

Sp21<-Sp1-Sp22

Sp12<-Sp2-Sp22

Sp22<-CovD_neg+(Sp1*Sp2)

CovD_pos~dunif(a1,b1)

CovD_neg~dunif(a2,b2)

a1<-(Se1- 1)*(1-Se2)

b1<-min(Se1,Se2)-(Se1*Se2)

a2<-(Sp1- 1)*(1-Sp2)

b2<-min(Sp1,Sp2)-(Sp1*Sp2)}

parameters <- c("Se1","Se2","Se3","Sp1","Sp2",

"Sp3","pi", "CovD_pos","CovD_neg")

Int1<-list(Se1=0.5,Se2=0.5,Se3=0.5,Sp1=0.5,Sp2=0.5,Sp3=0.5)

Int2<-list(Se1=0.7,Se2=0.7,Se3=0.7,Sp1=0.7,Sp2=0.7,Sp3=0.7)

Int3<-list(Se1=0.9,Se2=0.9,Se3=0.9,Sp1=0.9,Sp2=0.9,Sp3=0.9)

Ints <-list(Int1,Int2,Int3)

set.seed(1234);date()

jagsfit25 <- jags(data=data ,inits=Ints, n.chains=3,

parameters.to.save=parameters,

model.file=model,

n.thin=2,n.burnin = 10000,n.iter=100000);date()

print(jagsfit25)

a single intradermal tuberculin (SIT) test, interferon gamma (IFN-γ) assay, and a commercial ELISA test (M. bovis Ab)

Note:

Se1 = Se of a single intradermal tuberculin test

Sp1 = Sp of a single intradermal tuberculin test

Se2 = Se of interferon gamma assay

Sp2 = Sp of interferon gamma assay

Se3 = Se of a commercial ELISA test

Sp3 = Sp of a commercial ELISA test

pi = prevalence of the disease
