# Supplementary material for: Determination of the sensitivity and specificity of bovine tuberculosis screening tests in dairy herds in Thailand using a Bayesian approach
Source: BMC Vet Res. 2019 May 16;15:149. doi: 10.1186/s12917-019-1905-x (PMC6524330; doi:10.1186/s12917-019-1905-x)
Supplement: Supplementary file 2 — Checking of convergence of two conditionally dependent and one conditionally dependent with one population model using Gelman-Rubin diagnostic plots (DOCX 926 kb) [file 12917_2019_1905_MOESM2_ESM.docx]

**Supplementary 2**

Checking of convergence of two conditionally dependent and one conditionally dependent with one population model using Gelman-Rubin diagnostic plots


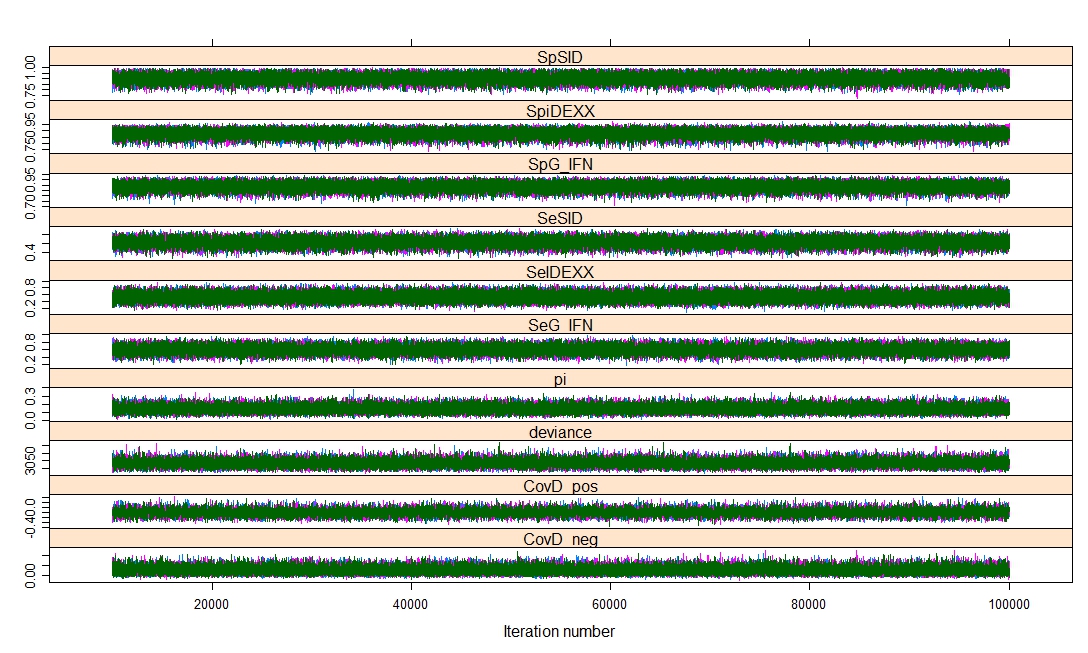


Figure 1 Trace plots of two conditionally dependent and one conditionally dependent with one population model


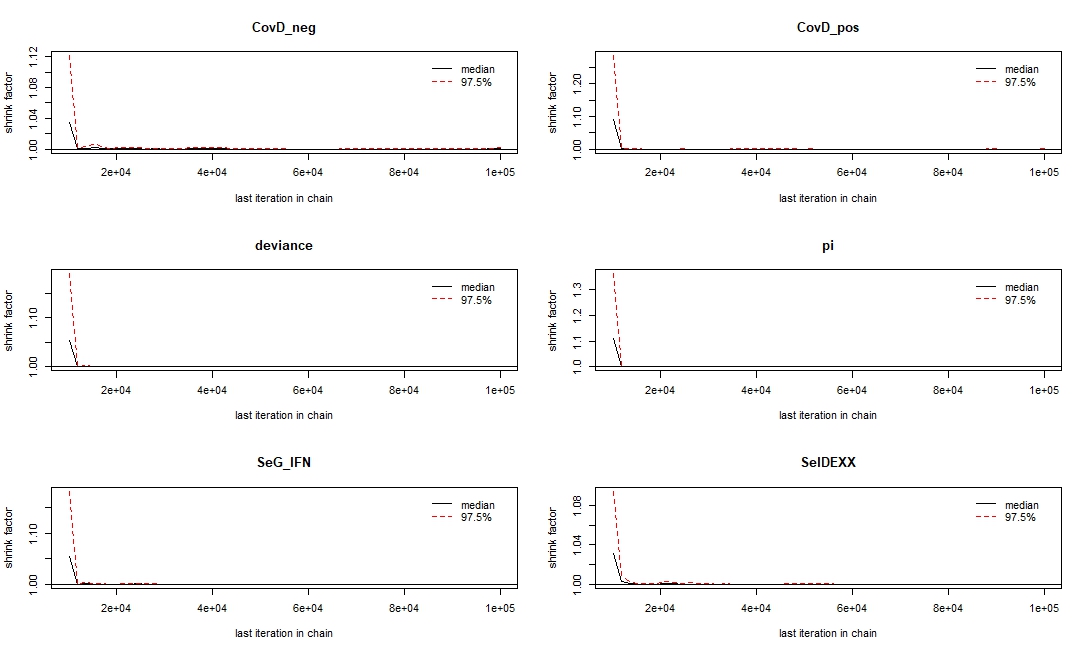


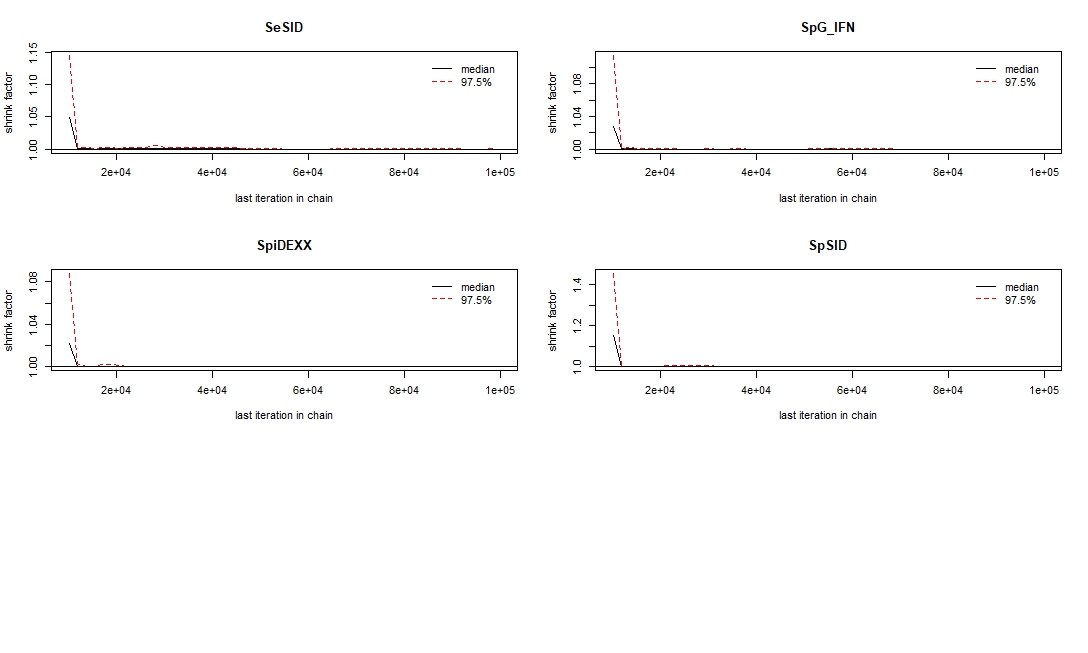
 Figure 2 Gelman-Rubin diagnostic plots of two conditionally dependent and one conditionally dependent with one population model
